# The association between egg consumption and metabolic health status in overweight and obese adolescents

**DOI:** 10.1038/s41598-023-30018-y

**Published:** 2023-02-16

**Authors:** Ali Tabatabaeyan, Keyhan Lotfi, Saeideh Mirzaei, Ali Asadi, Masoumeh Akhlaghi, Parvane Saneei

**Affiliations:** 1 Department of Clinical Nutrition, School of Nutrition and Food Science, Nutrition and Food Security Research Center, Students’ Research Committee, Isfahan University of Medical Sciences, Isfahan, Iran; 2 Department of Community Nutrition, School of Nutritional Sciences and Dietetics, Tehran University of Medical Sciences, Tehran, Iran; 3 Department of Community Nutrition, School of Nutrition and Food Science, Shiraz University of Medical Sciences, Shiraz, Iran; 4 Department of Exercise Physiology, School of Physical Education and Sport Sciences, University of Tehran, Tehran, Iran; 5 Department of Community Nutrition, School of Nutrition and Food Science, Nutrition and Food Security Research Center, Isfahan University of Medical Sciences, PO Box 81745-151, Isfahan, Iran; 6 Students’ Scientific Research Center, Tehran University of Medical Sciences, Tehran, Iran

**Keywords:** Cancer, Diseases, Health care, Health occupations, Medical research, Rheumatology

## Abstract

Existing evidence examining the relation between egg consumption and metabolic health of overweight/obese adolescents is scarce. We examined the association between egg consumption and metabolic status in Iranian overweight/obese adolescents. Using multistage cluster random sampling approach, overweight/obese adolescents (n = 203) with ages from 12 to 18 years old were selected for the present cross-sectional study. A validated 147-item food frequency questionnaire was adopted to determine usual dietary intakes. Blood pressure and anthropometric data and were assessed, and levels of lipid profile, insulin, and glucose were measured by collecting fasting blood samples. Participants were classified into metabolically healthy obese (MHO) or metabolically unhealthy obese (MUO) based on two methods of International Diabetes Federation (IDF) and the combination of IDF with Homeostasis Model Assessment Insulin Resistance (HOMA-IR). In total, 67 (33.0%) and 79 (38.9%) adolescents were classified as MUO based on IDF/HOMA and IDF definitions, respectively. Considering IDF criteria, the highest intake of egg was related to decreased chance of MUO, in crude (OR 0.22; 95% CI 0.10–0.48) and maximally-adjusted model (OR 0.25; 95% CI 0.10–0.59). Considering IDF/HOMA-IR criteria, similar results were obtained (crude model: OR 0.24; 95% CI 0.11–0.52; fully-adjusted model: OR 0.28; 95% CI 0.11–0.69). Stratified analyses found stronger relation among boys (vs. girls) and overweight (vs. obese) individuals. In conclusion, higher egg consumption was negatively related to decreased chance of being MUO in overweight/obese adolescents, especially in boys and overweight individuals, regardless of MUO definitions. Prospective studies are required to support our results.

## Introduction

Obesity has been known as an important public health problem during the present century^1^. Prior investigations have documented double increase in the overweight and obesity prevalence among children and adolescents in the last 30 years^2^. Similar to the growing trend in childhood obesity among developed countries; the existing data show that the developing countries have the same condition^3^. Highest rates of obesity have been reported among countries of the Latin America, North Africa, and Eastern Mediterranean region. World Health Organization (WHO) announced that the prevalence of childhood overweight in the Middle East is more considerable than some other developing countries^3^. According to a recent study, prevalence of obesity among 6–18 years old Iranians was estimated 13.58 and 10.15%, respectively^4^. Obesity could substantially lead to some adverse medical consequences such as dyslipidemia, hypertension, gastrointestinal diseases, cardiovascular diseases, type 2 diabetes, and some cancers^5,6^.

It should be noted that some overweight or obese children do not show the above-mentioned detrimental metabolic disorders; these individuals are named “metabolically healthy obese” (MHO)^7,8^. In other words, MHO refers to overweight or obese subjects who have optimal cardiometabolic health status and insulin sensitivity. In contrast, other subjects with worst cardiometabolic health status are named as “metabolically unhealthy obese” (MUO)^9,10^. The chance of being MUO might be increased by age^11^. However, some MHO subjects could keep their metabolic profile over time^12^. Some investigations suggested that the mechanisms involved in the origin of metabolic health status are insulin sensitivity, special fat distribution, reduced penetration of immune cells into adipose tissue, and therefore a pattern of metabolically beneficial cytokine and adipokine secretion^13,14^. Besides heredity, lifestyle features such as dietary habits, physical activity level, and their interactions might affect the metabolic health conditions^15,16^.

Several earlier studies have investigated the relation between food groups intake or macro/micronutrients and metabolic health status and resulted in contradictory findings^17,18^. A cross-sectional study showed that overweight/obese MHO females had a healthier dietary pattern with more intakes of fiber, vegetables, and lower saturated fat and dairy products intakes^18^. Another investigation on adults has suggested that MHO adults have less commercial sweets and more fish consumption^19^. A longitudinal study on 6,504 Iranian adults has evaluated the impact of more protein consumption on metabolic health and revealed a reduced risk of metabolic syndrome (MetS) following an additional frequency of protein intake^20^. An additional frequency consumption of egg was also associated with lower odds of MetS in this adult population^20^. Findings from 23,993 Korean adults demonstrated that the consumption of egg (4–6 times/week and 1 time/day) could lessen the risk of MetS compared to consuming less than one egg per month^21^. This review has also revealed that consuming ≥ 2 eggs/day was not related to MetS; only 4–7 times/week egg consumption was linked to decreased MetS risk^21^. According to our knowledge, most previous investigations on egg consumption and metabolic health conditions were done on American or European adults^19–21^ and the relationship was not studied in Iranians, especially in Iranian children or adolescents. So, the current study was designed to investigate egg consumption in relation to metabolic health status among Iranian adolescents. In order to have enough MUO cases, this study has been performed on overweight and obese adolescents.

## Methods and materials

### Participants

This cross-sectional investigation was done on 203 Iranian adolescents (101 boys/ 102 girls) aged 12–18 years’ old who were chosen from 16 schools of 5 major regions of the Isfahan city, Iran, by applying a multi-stage cluster random-sampling method. Height and body weight of all subjects were assessed and the body mass index (BMI) of all students was calculated. Then, students were classified into 3 groups of normal-weight, overweight, and obese, based on WHO growth curve (age- and sex-specific BMI percentiles)^22^. In this way, overweight and obese adolescents with different socioeconomic status (SES) were invited to participate in this investigation. Adolescents were excluded if they: (1) were mineral/vitamin supplements user or any medications that could have impact on lipid profile, blood pressure, body weight, or blood glucose, (2) adhering to a calorie-restricted (weight loss) diet, (3) had inherited or endocrine disturbances (including hypothyroidism, type 1 Cushing’s syndrome, and diabetes mellitus). An informed consent was signed by each adolescent and his/her parents. The protocol of this study was confirmed by Isfahan University of Medical Sciences local ethics committee (no. 2401108).

### Egg consumption assessment

Egg consumption was assessed using a validated 147-item semi-quantitative food frequency questionnaire (FFQ) which could assess usual dietary intake of each participant in the past year. This FFQ was validated among Iranian adolescents, previously^23^. Individuals’ foods consumption was questioned by a trained dietitian based on daily, weekly, or monthly intake. A standard portion size was used to evaluate the amount of egg and other foods intake. Then, the portion sizes were transformed to grams/day by using household measurements^24^. Eventually, the Nutritionist IV software that is linked to the USDA food composition database was used to determine nutrient intakes by entering the grams of egg and all other foods intake. Of note, some modifications for some Iranian foods were incorporated in the software to calculate the nutrient intake.

### Assessment of anthropometric and metabolic parameters

Two trained dietitians measured anthropometric indices of participants. The measurement of weight was done using a calibrated electronic scale (Seca Instruments, Germany) to the nearest 0.1 kg, without shoes and in minimal clothing. The standing height was measured using a stadiometer without shoes to the nearest 0.1 cm. BMI was calculated by weight (kg)/height squared (m^2^) formula. Then, adolescents with overweight (85th < BMI < 95th percentile) or obese (BMI > 95th percentile) (based on WHO age- and sex-standardized BMI cut-off points)^22^ were invited to the study. After normal breathing, the waist circumference (WC) was measured two times with an unstretched flexible tape without any pressure on the body surface at the midway between the lowest rib and the superior border of the iliac crest to the nearest 0.01 cm. The final WC for each subject was the mean of two measured values. A mercury sphygmomanometer was applied at the right arm to measure systolic blood pressure (SBP) and diastolic blood pressure (DBP). BP was assessed two times for each individual and mean of the two values was used as final SBP and DBP values. Biochemical indices were determined by collecting venous blood samples after a 12-h overnight fasting. The measurements of fasting blood glucose (FBG), triglycerides (TG), high-density lipoprotein cholesterol (HDL-c), and insulin levels were carried out by standard methods. Additionally, the value of the Homeostasis Model Assessment Insulin Resistance (HOMA-IR) was calculated through the following formula: HOMA-IR = [(fasting insulin (mU/L) × FBG (mmol/L)] /22.5.

### Metabolic health status assessment

Adolescents were categorized as MHO/MUO subjects by two distinct criteria. The first classification was based on the modified International Diabetes Federation (IDF) criteria^15^, by which adolescents were known to be MUO if they had 2 ≤ of the following risk factors: elevated TG (≥ 150 mg/dL), elevated FBG (≥ 100 mg/dL), high blood pressure (≥ 130/85 mmHg), and low HDL-c (< 40 mg/dL for the age of < 16 y, and < 50 mg/dL in girls/ < 40 mg/dL in boys for the age of ≥ 16 y); otherwise students were known as MHO. The second classification was based on absence or presence of insulin resistance, in addition to the IDF definition used for the first classification method^8,25^. In this procedure, students having insulin resistance (HOMO-IR ≥ 3.16, based on previous studies^8,25,26^) and 2 ≤ of the aforementioned risk factors were classified as MUO; whereas those without insulin resistance (HOMO-IR < 3.16) were classified as MHO.

### Assessment of other variables

Physical activity level of each participant was examined by the Physical Activity Questionnaire for Adolescents (PAQ-A) consisting of 9 questions about different aspects of physical activity in the preceding week^27^. The first 8 items of PAQ-A indicated the usual activity of individuals during the previous week and were scored from 1 to 5. Score 1 represented the lowest level of physical activity and score 5 represented the highest. Then, scores were added together. The last item of PAQ-A indicated unusual activities of individuals during the previous week. Subjects were categorized based on their total scores into sedentary or inactive (score < 2), low active (3 < score ≤ 2), moderately active (4 < score ≤ 3), and highly active (score ≥ 4). A validated demographic questionnaire was applied to assess participants’ socioeconomic status (SES) according to several items including: family size, having computers/laptops, having personal room, having cars in the family, parental education level, parental job, and traveling in the preceding year^28^. Then, a total score for SES was calculated. A pre-tested questionnaire was distributed among individuals to collect information about adolescents’ gender, age, medical history, and taking drugs or dietary supplements.

### Statistical analysis

Findings from previous studies among Iranian adolescents with overweight/obesity were used for sample size calculation, indicating that the prevalence of MUO was 40–60%^29,30^. Considering the type I error of 0.05, power of 80%, desired confidence interval (CI) of 95%, and accuracy (d) of 7%, a minimum of 188 adolescents was estimated to be required. First, we obtained energy-adjusted egg consumption based on residual method^16^. Then, study subjects were categorized according to tertiles of egg consumption (T_1_: < 20.09, T_2_: 20.09–33.01, T_3_: > 33.01 g/d). The qualitative and quantitative variables were respectively reported as frequency (percentage) and mean ± SD/SE. To assess the differences through tertiles of egg consumption, we used one-way analysis of variance (ANOVA) and chi-square test for continuous and categorical variables, respectively. The analysis of covariance (ANOVA) was used to calculate age, sex, and energy-adjusted dietary macro- and micro-nutrient intakes of participants. Multivariable logistic regression was used to examine tertiles of egg consumption in relation to MUO, and odds ratios (ORs) and 95% confidence intervals (CIs) for having MUO status were computed in crude and adjusted models. For the first model, gender, age, and energy intake were adjusted. More adjustments were done for SES and physical activity in the second model. For the last model, BMI was also adjusted in order to have an independent relation from obesity. We considered the first tertile of egg consumption as the reference category for all models. P for trend of ORs across increasing egg consumption tertiles was evaluated by considering tertiles of egg consumption as a continuous variable. Stratified analyses by BMI (overweigh vs. obese) and sex categories (girls vs. boys) were performed. SPSS software version 26 (IBM, Chicago, IL) was used for all analyses. *P* values < 0.05 (two-tailed) were considered as statistically significant.

### Ethical approval and consent to participate

The study procedure was performed according to declaration of Helsinki guideline. All participants provided informed written consent. The study protocol was approved by the local Ethics Committee of Isfahan University of Medical Sciences.

### Consent to participate

Informed consent was obtained from all participants involved in the study.

## Results

A total of 203 adolescents were included in the current study. Mean age, weight, and BMI of participants were 13.98 ± 1.61, 73.48 ± 11.60, and 27.35 ± 3.24, respectively; 49.8% of them (n = 101) were boys. General characteristics and cardio-metabolic risk factors of study subjects across tertiles of energy-adjusted egg consumption are represented in Table 1. There was no significant difference in age, BMI, weight, height, and waist circumference of individuals at different categories of egg consumption. Adolescents with the highest of egg consumption were more active than those with the lowest. Also, individuals in the top tertile of egg consumption compared to those in the first bottom tertile had lower FBS, triglycerides, HOMA-IR, and insulin, and higher HDL-c.

**Table 1:** General characteristics and cardiometabolic factors of study participants across tertiles of egg consumption.^1^

	Tertiles of egg consumption			
	T1 (n = 67)	T2 (n = 68)	T3 (n = 68)	*P* value^2^
Range	(< 20.09 g/d)	(20.09–33.01 g/d)	(> 33.01 g/d)	
Sex, n (%)				0.64
Boys	32 (47.8)	32 (47.1)	37 (54.4)	
Girls	35 (52.2)	36 (52.9)	31 (45.6)	
Age (year)	13.63 ± 1.41	14.13 ± 1.62	14.16 ± 1.73	0.09
Weight (kg)	74.84 ± 11.35	72.73 ± 10.71	72.88 ± 12.70	0.50
Height (cm)	163.41 ± 7.67	163.62 ± 6.97	163.86 ± 9.14	0.94
BMI (kg/m^2^)	27.98 ± 3.57	27.07 ± 2.74	27.02 ± 3.30	0.15
Waist circumference (cm)	91.02 ± 6.72	89.50 ± 7.21	90.50 ± 9.60	0.53
Physical activity levels, n (%)				< 0.001
Sedentary	63 (94.0)	58 (85.3)	45 (66.2)	
Active	4 (6.0)	10 (14.7)	23 (33.8)	
Socioeconomic status, n (%)				0.53
Low	21 (31.3)	17 (25.0)	21 (30.9)	
Medium	26 (38.8)	36 (52.9)	28 (41.2)	
High	20 (29.9)	15 (22.1)	19 (27.9)	
Systolic blood pressure (mmHg)	114.73 ± 21.86	112.99 ± 16.65	110.43 ± 16.05	0.39
Diastolic blood pressure (mmHg)	73.25 ± 15.51	73.87 ± 10.13	73.37 ± 7.07	0.94
Fasting blood glucose level (mg/dL)	100.61 ± 9.57	98.37 ± 7.64	95.46 ± 7.50	0.01
Insulin (μUI/mL)	22.68 ± 12.57	21.71 ± 15.40	16.90 ± 8.40	0.02
HOMA-IR index	5.68 ± 3.40	5.34 ± 3.83	4.04 ± 2.19	0.01
Triglycerides (mg/dL)	139.21 ± 71.63	118.56 ± 59.71	108.34 ± 65.1	0.02
HDL-c (mg/dL)	43.22 ± 7.87	44.29 ± 7.05	46.93 ± 8.43	0.02

*HOMA* Homeostasis Model Assessment Insulin Resistance, *HDL-c* high-density lipoprotein cholesterol, *BMI* Body Mass Index.

^1^Values are mean ± SD for continuous and percentage for categorical variables.

^2^*P* values were obtained from one-way ANOVA and Chi-square test for continuous and categorical variables, respectively.

Dietary intakes of study participants across tertiles of energy-adjusted egg consumption are provided in Table 2. Individuals with the highest egg consumption category compared with the lowest one consumed more fat, protein, monounsaturated fatty acids, cholesterol, saturated fatty acids, vitamin C, riboflavin, vitamin B12, folate, vitamin B6, magnesium, potassium, zinc, and total dietary fiber and less carbohydrates.

**Table 2:** Dietary intakes (energy and macro/micro nutrients) of study participants across tertiles of egg consumption.^1^

	Tertiles of egg consumption			
	T1 (n = 67)	T2 (n = 68)	T3 (n = 68)	*P* value^2^
Range	(< 20.09 g/d)	(20.09–33.01 g/d)	(> 33.01 g/d)	
Energy, kcal	2928.38 ± 67.16	2865.11 ± 66.03	2856.24 ± 66.34	0.71
Protein, % of energy	13.52 ± 0.23	14.08 ± 0.22	15.29 ± 0.23	< 0.001
Carbohydrate, % of energy	59.39 ± 0.61	59.42 ± 0.60	56.07 ± 0.60	< 0.001
Fat, % of energy	28.51 ± 0.63	27.88 ± 0.62	30.13 ± 0.62	0.03
Cholesterol, mg	201.78 ± 8.10	263.77 ± 7.96	379.46 ± 8.00	< 0.001
SFA, gr	26.33 ± 0.70	26.42 ± 0.68	29.29 ± 0.69	0.003
MUFA, gr	26.62 ± 0.82	26.24 ± 0.81	29.77 ± 0.81	0.004
PUFA, gr	29.34 ± 0.98	27.77 ± 0.97	28.34 ± 0.97	0.52
Vitamin C, mg	123.67 ± 7.18	128.82 ± 7.06	148.24 ± 7.09	0.04
Vitamin A, RAE	1059.70 ± 77.90	1059.05 ± 76.52	1202.81 ± 76.90	0.31
Thiamin, mg	2.68 ± 0.03	2.67 ± 0.03	2.57 ± 0.03	0.06
Riboflavin, mg	2.06 ± 0.06	2.19 ± 0.06	2.64 ± 0.06	< 0.001
Niacin, mg	27.81 ± 0.42	28.01 ± 0.41	26.88 ± 0.42	0.13
Vitamin B6, mg	1.52 ± 0.05	1.57 ± 0.04	1.77 ± 0.05	0.001
Vitamin E, mg	31.55 ± 1.42	29.95 ± 1.40	29.58 ± 1.40	0.58
Folate, mcg	297.22 ± 11.83	290.79 ± 11.63	361.57 ± 11.68	< 0.001
Vitamin B12, mcg	3.81 ± 0.17	4.27 ± 0.16	5.20 ± 0.17	< 0.001
Magnesium, mg	274.20 ± 7.37	272.62 ± 7.24	317.52 ± 7.27	< 0.001
Zinc, mg	9.96 ± 0.25	9.99 ± 0.25	11.95 ± 0.25	< 0.001
Selenium, mcg	0.10 ± 0.004	0.09 ± 0.003	0.09 ± 0.003	0.07
Total fiber, gr	18.83 ± 0.59	18.78 ± 0.58	20.71 ± 0.58	0.03
Sodium, mg	4198.04 ± 140.72	3747.43 ± 138.23	4023.85 ± 138.90	0.07
Potassium, mg	3155.37 ± 106.21	3208.08 ± 104.33	3766.72 ± 104.84	< 0.001

*SFA* saturated fatty acids, *MUFA* monounsaturated fatty acids, *PUFA* polyunsaturated fatty acids.

^1^Values are mean ± SE. Energy intake and macronutrients were adjusted for age and gender; all other values were adjusted for age, gender and energy intake.

^2^*P* values were obtained from ANCOVA test.

**Figure 1:**
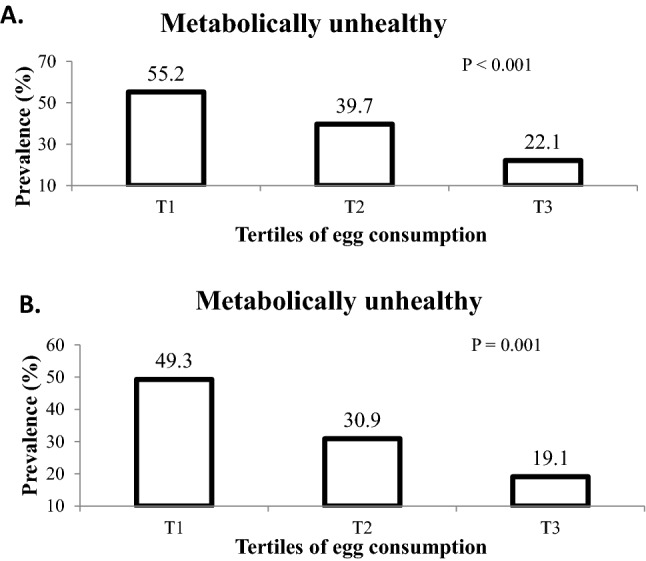
Prevalence of MUO in tertiles of egg consumption. (**A**) MUO based on IDF definition. (**B**) MUO Based on IDF/HOMA-IR IDF definition. Values are percentage of individuals with a metabolically unhealthy phenotype in tertiles of egg consumption. *P* values were obtained from chi-square test.

The prevalence of MUO (based on IDF and IDF/HOMA-IR criteria) at different categories of egg consumption are shown in Fig. 1. According to the IDF criteria, adolescents in the top third tertile of egg consumption have lower prevalence of MUO, compared to the first tertile (22.1 vs. 55.2%, *P* < 0.001). Similarly, considering IDF/HOMA-IR criteria, the prevalence of MUO in the last tertile of egg consumption was lower than the first tertile (19.1 vs. 49.3%, *P* = 0.001).

Multivariate adjusted odds ratio (OR) and 95% confidence interval (CI) for MUO across tertiles of energy-adjusted egg consumption are presented in Table 3. Considering IDF criteria, adolescents in the top category of egg consumption compared to the bottom category had a 78% lower chance for MUO, in the crude model (OR 0.22; 95% CI 0.10–0.48); the results remained unchanged after adjusting potential confounders (OR 0.25; 95% CI 0.10–0.59). Considering IDF/HOMA-IR criteria, adolescents in the third tertile of egg consumption compared to the first tertile had a 76% lower odds for MUO, in the crude model (OR 0.24; 95% CI 0.11–0.52); after adjustment for confounding factors, those with the highest egg consumption had a 72% lower possibility for MUO (OR 0.28; 95% CI 0.11–0.69), compared to the reference category.

**Table 3:** Multivariate adjusted odds ratio (OR) and 95% confidence interval (CI) for MUO across tertiles of egg consumption.^1^

	Tertiles of egg consumption			
	T1 (n = 67)	T2 (n = 68)	T3 (n = 68)	P-trend
MUO phenotype based on IDF criteria				
Cases (n)	37	27	15	
Crude	1.00	0.53 (0.26, 1.05)	0.22 (0.10, 0.48)	< 0.001
Model 1^2^	1.00	0.49 (0.24, 1.02)	0.21 (0.09, 0.47)	< 0.001
Model 2^3^	1.00	0.51 (0.24, 1.09)	0.24 (0.10, 0.56)	0.01
Model 3^4^	1.00	0.55 (0.25, 1.18)	0.25 (0.10, 0.59)	0.01
MUO phenotype based on HOMA-IR criteria				
Cases (n)	33	21	13	
Crude	1.00	0.46 (0.22, 0.93)	0.24 (0.11, 0.52)	< 0.001
Model 1^2^	1.00	0.43 (0.20, 0.92)	0.23 (0.10, 0.53)	0.01
Model 2^3^	1.00	0.45 (0.20, 0.98)	0.26 (0.10, 0.63)	0.01
Model 3^4^	1.00	0.50 (0.22, 1.10)	0.28 (0.11, 0.69)	0.01

^1^All values are odds ratios and 95% confidence intervals.

^2^Model 1: Adjusted for sex, age, and energy intake.

^3^Model 2: Model 1 + further adjustments for SES and physical activity.

^4^Model 3: Model 2 + more adjustment for BMI.

Table 4 shows multivariate adjusted OR and 95% CI for MUO across tertiles of energy-adjusted egg consumption, stratified by BMI categories. Considering IDF definition, egg consumption was significantly associated with reduced odds of MUO in both overweight and obese groups. According to IDF/HOMA-IR criteria, only in overweight group, the highest category of egg consumption compared to the lowest category was associated with decreased odds of MUO, in fully-adjusted model. Multivariate adjusted OR and 95% CI for MUO across tertiles of energy-adjusted egg consumption, stratified by sex are provided in Table 5. After adjustment for potential confounders, higher egg consumption was related to decreaseded odds of MUO in both sex groups, when we considered IDF criteria to define MUO. Based on IDF/HOMA-IR definition, in fully-adjusted model, the relationship was significant only among boys.

**Table 4:** Multivariate adjusted odds ratio (OR) and 95% confidence interval (CI) for MUO across tertiles of energy-adjusted egg consumption, stratified by BMI categories.^1^

	Tertiles of egg consumption			
	T1	T2	T3	P-trend
MUO phenotype based on IDF criteria				
Overweight (cases/participants)	12/27	11/36	4/41	
Crude	1.00	0.55 (0.19, 1.55)	0.17 (0.05, 0.57)	0.01
Model 1^2^	1.00	0.48 (0.16, 1.44)	0.18 (0.05, 0.67)	0.01
Model 2^3^	1.00	0.34 (0.98, 1.19)	0.18 (0.04, 0.73)	0.01
Obese (cases/participants)	25/40	16/32	10/27	
Crude	1.00	0.60 (0.23, 1.54)	0.35 (0.12, 0.96)	0.04
Model 1^2^	1.00	0.56 (0.21, 1.51)	0.26 (0.08, 0.78)	0.01
Model 2^3^	1.00	0.65 (0.23, 1.81)	0.25 (0.07, 0.81)	0.02
MUO phenotype based on IDF /HOMA-IR criteria				
Overweight (cases/participants)	10/27	7/36	3/41	
Crude	1.00	0.41 (0.13, 1.27)	0.13 (0.03, 0.55)	0.01
Model 1^2^	1.00	0.33 (0.10, 1.14)	0.14 (0.03, 0.64)	0.01
Model 2^3^	1.00	0.25 (0.06, 0.99)	0.15 (0.03, 0.74)	0.01
Obese (cases/participants)	23/40	14/32	10/27	
Crude	1.00	0.57 (0.22, 1.46)	0.43 (0.16, 1.18)	0.09
Model 1^2^	1.00	0.55 (0.20, 1.47)	0.33 (0.11, 0.99)	0.04
Model 2^3^	1.00	0.61 (0.22, 1.70)	0.33 (0.10, 1.04)	0.06

^1^All values are odds ratios and 95% confidence intervals.

^2^Model 1: Adjusted for sex, age, and energy intake.

^3^Model 2: Model 1 + further adjustments for SES and physical activity.

**Table 5:** Multivariate adjusted odds ratio (OR) and 95% confidence interval (CI) for MUO across tertiles of energy-adjusted egg consumption, stratified by sex.^1^

	Tertiles of egg consumption			
	T1	T2	T3	P-trend
MUO phenotype based on IDF criteria				
Girl (cases/participants)	20/35	15/36	7/31	
Crude	1.00	0.53 (0.20, 1.37)	0.21 (0.07, 0.64)	0.01
Model 1^2^	1.00	0.57 (0.20, 1.61)	0.24 (0.07, 0.78)	0.01
Model 2^3^	1.00	0.58 (0.19, 1.75)	0.28 (0.08, 0.98)	0.04
Model 3^4^	1.00	0.59 (0.19, 1.79)	0.29 (0.08, 0.98)	0.04
Boy (cases/participants)	17/32	12/32	8/37	
Crude	1.00	0.52 (0.19, 1.43)	0.24 (0.08, 0.69)	0.01
Model 1	1.00	0.45 (0.16, 1.29)	0.19 (0.06, 0.61)	0.01
Model 2	1.00	0.46 (0.15, 1.41)	0.22 (0.06, 0.77)	0.01
Model 3	1.00	0.52 (0.17, 1.61)	0.24 (0.06, 0.88)	0.03
MUO phenotype based on IDF /HOMA-IR criteria				
Girl (cases/participants)	17/35	10/36	5/31	
Crude	1.00	0.40 (0.15, 1.09)	0.20 (0.06, 0.65)	0.01
Model 1	1.00	0.48 (0.16, 1.44)	0.27 (0.07, 0.94)	0.03
Model 2	1.00	0.53 (0.17, 1.70)	0.35 (0.09, 1.34)	0.11
Model 3	1.00	0.56 (0.17, 1.80)	0.36 (0.09, 1.39)	0.13
Boy (cases/participants)	16/32	11/32	8/37	
Crude	1.00	0.52 (0.19, 1.43)	0.27 (0.09, 0.78)	0.01
Model 1	1.00	0.43 (0.15, 1.25)	0.21 (0.07, 0.68)	0.01
Model 2	1.00	0.42 (0.13, 1.32)	0.24 (0.07, 0.85)	0.02
Model 3	1.00	0.47 (0.14, 1.50)	0.27 (0.07, 0.96)	0.04

^1^All values are odds ratios and 95% confidence intervals.

^2^Model 1: Adjusted for age and energy intake.

^3^Model 2: Model 1 + further adjustments for SES and physical activity.

^4^Model 3: Model 2 + more adjustment for BMI.

## Discussion

We found that being MUO is less likely to occur in Iranian adolescents who consumed higher amounts of egg. The relation was found to be stronger in boys (vs. girls) and overweight (vs. obese) individuals. The observed association was independent from the applied definitions for MUO and potential confounders. This study was the first investigation assessed the relationship between egg consumption and metabolic health condition in adolescents in a Middle Eastern country.

Overweight or obese subjects with MHO status have a relatively proper health condition. Maintaining this condition is worthwhile to prevent obesity-related complications. Our findings suggested that egg consumption could play a positive role in keeping MHO profile over time. Therefore, children and adolescents could be clinically advised to consume more egg, which can provide a source of high-value protein and nutrients and help to maintain their metabolic health status.

Some previous studies have investigated egg consumption in relation to metabolic health, but their results were contradictory. A cross-sectional study on 1,008 Irish middle-age men and 1,039 women showed diet as an important determinant of the obesity phenotype and general health condition^31^. In a cross-sectional study on adults, Wang et al. declared that egg consumption was inversely related to metabolic syndrome, particularly in female individuals^32^. In contrast, the HELENA study on Spanish adolescents revealed that egg consumption was not related to blood pressure, insulin resistance, adiposity, lipid profile, and cardio-respiratory fitness^33^. A review study analyzed cross-sectional data from 23,993 Korean adults and reported that consuming egg 4–7 times per week could be related to a decreased risk of metabolic syndrome, but consuming ≥ 2 eggs/day was not related to a decreased risk^21^.

Prospective investigations have also achieved different findings with regard to egg consumption and metabolic risk factors. In an interventional study on 955 healthy students, co-supplementation with 50 g of egg and 200 g of milk along with a normal diet in comparison to the normal diet for 2 years had positive effects on growth and physical fitness in poor rural Chinese children^34^. Furthermore, Isfahan Cohort Study (ICS) conducted on 6504 middle-age adults followed for 11.25 years revealed that each additional frequency consumption of egg was substantially related to a 21% lowered risk of MetS^20^. Additionally, a recently published nested case–control study on 3401 cardiovascular disease (CVD) cases and 1377 controls revealed that moderate egg consumption in the Chinese population had protective effects on CVD markers^35^. A recent crossover trial on Australian adults has also showed that after eating egg for breakfast, overweight and obese individuals had a lower energy intake at lunch meal in comparison to those who consumed a cereal breakfast; this trial suggested that egg consumption may help overweight and obese subjects manage their food intake to lose weight^36^. In contrast, in another crossover trial conducted on children and adolescents, lunch meal intake after a breakfast with egg was not significantly different from a breakfast with bagel^37^. Different findings on the relation between egg consumption and metabolic health status might be explained by various study designs, populations, assessments tools, statistical analyses, and adjustments for confounders.

Recent in-vitro and in-vivo investigations have suggested that bioactive peptides derived from egg proteins could display multiple physiological activities, including antihypertensive (angiotensin I-converting enzyme (ACE) inhibitory), immunomodulatory, anticancer, antioxidant, antimicrobial, antidiabetic, and anti-obesogenic activities^38,39^. These bioactive peptides are protein fragments that can directly be absorbed through the gut and enter the blood circulation. Experimental studies have reported promising health effects for these egg-based peptides, especially in obesity^38,39^. The administration of egg white hydrolysed with pepsin for 12 weeks in Zucker rats could significantly decrease obesity-related disorders, including epididymal adipose tissue, serum free fatty acids and tumor necrosis factor-alpha in the obese animals^40^. These results could support anti-obesogenic impacts of egg and somehow the finding of the current investigation in adolescents.

Egg is rich source of high quality protein, minerals, vitamins, as well as cholesterol, unsaturated and saturated fats^41^. Therefore, despite having high amounts of cholesterol, some beneficial nutrients of egg might be responsible for the inverse relation between egg intake and MUO. Egg white protein and lactic-fermented egg white (a simply consumable form of egg white) would be beneficial for both preventing and alleviating metabolic disorders^42^. A study comparing the effect of whole egg with yolk-free alternatives on lipoprotein and plasma carotenoids among individuals with MetS demonstrated that whole egg could have beneficial impact on plasma carotenoid as well as saturation of LDL-c and HDL-c lipoproteins with zeaxanthin and lutein. These factors may affect cardio-metabolic risk factors^43^. Two more other randomized controlled trials have reported that whole eggs have positive effects on metabolic syndrome components by affecting plasma HDL-c lipoprotein saturation^43,44^. An interventional study conducted on patients with metabolic syndrome showed that egg yolk consumption for 12 weeks did not have negative effects on the inflammatory biomarkers. Furthermore, it has been suggested that inflammation might improve when egg consumption is accompanied by a carbohydrate restriction diet and weight loss. Therefore, the nutrient contents of the whole egg may be useful for metabolic risk factors due to their role in the balancing of inflammation^44^.

The present study has several strengths. First, this was one of the first studies investigated the relationship between egg consumption and metabolic status in obese and overweight adolescents. Second, we studied a sample size of both boys and girls, which made the results generalizable to both sexes. Third, we considered some possible confounders in our analyses and the results were independent from these potential confounders. However, some limitations should be noticed while interpreting our findings. As a major limitation of this cross-sectional investigation, we are not able to infer a causal association between egg consumption and being MUO. In addition, the dietary assessment was assessed via a self-reported FFQ; so, measurement errors might have occurred and misclassification of study participants might be a concern, even though our assessment has been carried out by a validated FFQ. It is also noteworthy that despite considering some potential confounders, we could not eliminate the effect of the remaining confounders such as birth weight, pubertal status and sleep behaviors.

In conclusion, we found higher egg consumption to be associated with decreased chance of MUO among Iranian overweight and obese adolescents, especially in boys and overweight individuals. This inverse association was independent from MUO definitions. Egg consumption could play a positive role in health status of adolescents. Further prospective studies are needed to confirm these findings.

## Data Availability

The data that support the findings of this study are available from the corresponding author upon reasonable request.

## Abbreviations

BMI: Body mass index
TG: Triglycerides
FBG: Fasting blood glucose
HDL-c: High density lipoprotein cholesterol
DBP: Diastolic blood pressure
SBP: Systolic blood pressure
WC: Waist circumference
FFQ: Food frequency questionnaire
IDF: International Diabetes Federation
MHO: Metabolically healthy obesity
MUO: Metabolically unhealthy obesity
HOMA-IR: Homeostasis Model Assessment Insulin Resistance
SES: Socioeconomic status
WHO: World Health Organization
PAQ-A: Physical Activity Questionnaire for Adolescents
SFA: Saturated fatty acids
MUFA: Mono unsaturated fatty acids
SCFA: Short chain fatty acids
ANOVA: Analysis of variance
ANCOVA: Analysis of covariance
95% CI: 95% Confidence interval
OR: Odds ratios
SPSS: Statistical package for the social sciences
